# A Multimodal Feature Fusion-Based Deep Learning Method for Online Fault Diagnosis of Rotating Machinery

**DOI:** 10.3390/s18103521

**Published:** 2018-10-18

**Authors:** Funa Zhou, Po Hu, Shuai Yang, Chenglin Wen

**Affiliations:** 1 School of Computer and Information Engineering, Henan University, Kaifeng 475004, China; yangshuai2711@163.com; 2 School of Automatic, Hangzhou Dianzi University, Hangzhou 310018, China; wencl@hdu.edu.cn

**Keywords:** fault diagnosis, deep learning, multimodal feature, DNN, feature fusion

## Abstract

Rotating machinery usually suffers from a type of fault, where the fault feature extracted in the frequency domain is significant, while the fault feature extracted in the time domain is insignificant. For this type of fault, a deep learning-based fault diagnosis method developed in the frequency domain can reach high accuracy performance without real-time performance, whereas a deep learning-based fault diagnosis method developed in the time domain obtains real-time diagnosis with lower diagnosis accuracy. In this paper, a multimodal feature fusion-based deep learning method for accurate and real-time online diagnosis of rotating machinery is proposed. The proposed method can directly extract the potential frequency of abnormal features involved in the time domain data. Firstly, multimodal features corresponding to the original data, the slope data, and the curvature data are firstly extracted by three separate deep neural networks. Then, a multimodal feature fusion is developed to obtain a new fused feature that can characterize the potential frequency feature involved in the time domain data. Lastly, the fused new feature is used as the input of the Softmax classifier to achieve a real-time online diagnosis result from the frequency-type fault data. A simulation experiment and a case study of the bearing fault diagnosis confirm the high efficiency of the method proposed in this paper.

## 1. Introduction

Large-scale intelligent production processes are becoming more and more complicated, as they are closely connected with each other. Mechanical equipment is a kind of key equipment for some intelligent production processes, since it can effectively avoid causality accidents in intelligent production processes [1,2,3,4,5,6,7,8]. However, real-time fault diagnosis with high accuracy for rotating machinery is still a challenge for safety guarantee, since most existing methods use the Fourier transform as preprocessing tool, which uses the frequency spectral as the source data. 

In the most recent three decades, research on fault diagnosis of key equipment in intelligent production processes has attracted extensive attention from academic and engineering researchers [6,9,10,11,12,13]. In general, three classes of fault diagnosis methods are now developed: fault diagnosis methods based on a physical model, a fault diagnosis method based on knowledge, and a data-driven-based method. However, precise physical model requirements limit its application in the field of fault diagnosis for complex mechanical equipment, and the processing of a quantity of prior knowledge limits its inference validation. Nowadays, data-driven techniques are widely applied in fault diagnosis since only historical data is required for establishing a fault diagnosis model [14,15,16]. The principal component analysis (PCA), support vector machine (SVM), and artificial neural network (ANN) are the most commonly used data-driven techniques for fault diagnosis [17,18,19,20,21,22]. However, methods based on statistical feature extraction, such as PCA, have certain limitations, including a requirement for statistical distribution of the data. For example, the PCA-based method can only detect faults but not diagnose faults. Compared with the statistical feature extraction method, the machine learning-based method has the advantages of high classification accuracy, strong robustness to noise, and fault tolerance. As a machine learning method, SVM can be used for fault classification. Some scholars put forward the use of signal processing feature extraction methods, combined with machine learning methods for mechanical equipment fault diagnosis. Yang et al. [23] extracted frequency-domain features as the data sources of SVM to detect mechanical faults. Although SVM performs well in binary classification, it has low learning efficiency in multi-classification and large sample processing [24,25]. In addition, expert experience is required to choose suitable kernel functions and scale parameters. The ANN has the characteristics of more accurate learning and stronger robustness when a large number of training samples are available, and feature pre-extraction of ANN can be used to further improve diagnosis efficiency [26,27,28]. Wang et al. [29] used wavelet packet transform (WPT) to extract the non-stationary characteristics of the bearing’s vibration signal as the pre-extracted feature of ANN, which has achieved good fault classification accuracy. However, it is difficult to effectively extract features involved in high-dimensional and non-steady data by using shallow learning methods that suffer the following deficiencies: (1) The convergence of ANN easily falls into a local optimum; thus, it cannot effectively characterize the signal characteristics; (2) low efficiency of ANN learning is another shortcoming of ANN. Compared with shallow learning, deep learning (DL) can perform feature extraction well and effectively handle nonlinear big data [30,31,32]. Therefore, DL is a promising tool for fault diagnosis [33,34,35,36]. 

As one of the most popular machine learning methods in the world, DL methods are widely used in many fields [37]. Deep neural network (DNNs) are a kind of DL that is comprised by unsupervised layer-by-layer greedy training and global parameter tuning based on the back propagation (BP) algorithm, which can effectively avoid local optimal problems as well as extracting deep features that are potentially involved in the data. In 2006, Science published Hinton’s article “Reducing the Dimensionality of Data with Neural Networks” to further discuss DNNs, and DL has once again become a hotspot research field. DNNs can obtain a more abstract high-level representation of the input data by combining lower-level features, which are nonlinear transformations of its previous layer. Attributed to this advantage, DNNs can extract complex nonlinear deep features without artificial feature selection [38]. Due to its excellent feature extraction capabilities, DL quickly attracted the attention of fault diagnosis researchers. Lu et al. used the good feature extraction ability of DNNs to successfully diagnose unknown types of bearing faults timely and effectively [39]. Jia et al. [40] used DNNs to detect the health status of rolling shaft bearings. The method that they developed can adaptively extract potential fault characteristics from nonlinear and noise-polluted observation data, so the accuracy of the diagnosis method is superior to other shallow learning-based methods. Gan et al. [41] used wavelet packet decomposition to develop a hierarchical DNN-based fault diagnosis method to more accurately diagnose faults in the time-frequency domain, since it can overcome the overlapping problem caused by noise and other disturbances. However, the above-mentioned DNN fault diagnosis methods do not take the data’s multimodal features into consideration. Li et al. [42] proposed a multimodal deep support vector classification for gearbox fault diagnosis, where Gaussian–Bernoulli Deep Boltzmann Machines (GDBMs) are used to extract the feature of the vibration signal in the time domain, the frequency domain, and the wavelet domain separately and then integrate them. However, the above methods are not real-time fault diagnosis methods, since the Fourier transform or wavelet transform is used to obtain the frequency-domain data from a full time domain. Pan et al. [43] proposed a LiftingNet to learn features adaptively from raw mechanical data without prior knowledge, by adopting the idea of a convoluted neural network and a second generation wavelet transform. In the case where considerable noise and randomness are involved in the observation data, the LiftingNet-based diagnosis method can achieve a reliable fault classification result, since the size of the convolution kernel can be selected as different sizes. Zhao et al. [44] proposed an improved deep residual network (DRN) with dynamically weighted wavelet coefficients (DRN + DWWC) to use the frequency band containing the most potential fault information as the dynamic weighting layer, of which weight is adjustable for more accurate feature extraction of different faults. Experiment analysis shows that the DRN + DWWC-based method is superior to other methods. 

Nevertheless, even the existing DNN-based method diagnosis method in the time domain is not good for the Tennessee Eastman (TE) process [45], as it usually cannot obtain high fault diagnosis accuracy for mechanical systems. The main reason is that most of the measured signals of rotating machinery are vibration signals with fault features that are extracted from abnormal vibration signals in the time domain are very small, and the fault feature extracted in the frequency domain is significant [46,47,48,49,50,51,52]. In this paper, we call this type of fault a “frequency-type fault”. The existing multimodal DL method considers the multimodality of features, but it cannot guarantee real-time performance. Therefore, the accuracy of real-time diagnosis in the time domain cannot be guaranteed. If such a fault cannot be diagnosed online in time, it can lead to faster machinery damage, which will result in irreparable damage or serious safety incidents. Therefore, it is of much practical significance to carry out research on the real-time diagnosis of frequency-type faults.

**Remark** **1.** *“Frequency-type fault” is only a conceptual description. It does not imply that a frequency fault is added into the system. In this paper, it is defined as a type of fault, of which fault feature extracted in the frequency domain is large, but a fault feature extracted in the time domain that is small. This type of fault can thus be usually well diagnosed in the frequency domain, but accurate and real-time fault diagnosis for this type of fault in the time domain cannot be guaranteed.*

There are many zero-crossing values in the bearing data, since they are mostly vibration signals [53]. This means that it is common for bearings to suffer frequency-type faults, and DNNs are unsuitable for distinguishing frequency-type faults based on the amplitude value of the observation data only, for the reason that multimodal differential features, such as slopes and curvatures, are potentially involved in the vibration signal. On the other hand, the differential geometry feature of the vibration signal, such as slopes and curvatures, can reveal the frequency-varying feature in the time domain. Research on accurate and real-time fault diagnosis methods for bearing fault diagnosis is required for developing an efficient multimodal feature fusion mechanism to fuse different trend features involved in the amplitude signal, slope signal, and curvature signal. Thus, frequency-type fault features characterized by different dynamic trends can be well extracted in the time domain. 

To improve the efficiency of a DL-based method for online diagnosis of a frequency-type fault, a multimodal differential geometric feature fusion-based DNN (DGFFDNN) fault diagnosis method was developed to fuse the abnormal frequency feature extracted in the time domain by combining the characterized dynamic trend features involved in multimodal differential geometric characteristics via three separate DNNs corresponding to the amplitude signal, the slope signal and the curvature signal. The fused multimodal features are then used as an input in the Softmax classifier to obtain an accurate online frequency-type diagnosis result. 

The remainder of this paper is organized as follows: Section 2 is a review of DL theory. In Section 3, an online original DGFFDNN fault diagnosis method is developed. In Section 4, the validity of the proposed fault diagnosis method is tested through experiments and simulation analysis. Section 5 contains conclusions and suggests future work. 

## 2. Review of Deep Learning Theory

DL is a feature learning method that can transform data from the raw data space into a higher-level feature space, and more abstract expressions of the data can be obtained through simple nonlinear models. Very complex functions can also be learned with a combination of enough layers of conversion. At present, DL is widely used in many fields [37]. For example, convolutional neural networks have achieved good performance in image processing. Recursive neural networks have significant effects in serialization tasks such as financial data prediction. 

**Figure 1:**
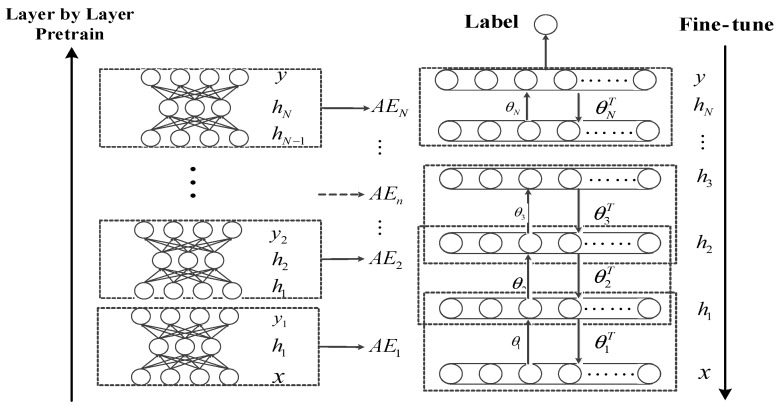
The structure of a deep neural network (DNN).

DNNs are a kind of DL method and have now been used in fault diagnosis as well as image processing and natural language processing. The training process of the DNN network shown in Figure 1. The DNN can be simply constructed by stacking multiple Auto-Encoder (AE) layers. The bottom-up unsupervised learning algorithm is used to roughly extract features layer-by-layer, and the entire network parameters can be fine-tuned with a supervised learning algorithm. By multilayer nonlinear transformation, low-level features are combined to form a more abstract high-level feature expression, which can extract features that are involved in the data, without relying on manual feature selection. Then, the DNN is fine-tuned by supervised learning to optimize the network parameters of the feature extraction network. The training mechanism of DNN is an advantage for effectively mining the fault features involved in vibration signals of the mechanical device. 

**Figure 2:**
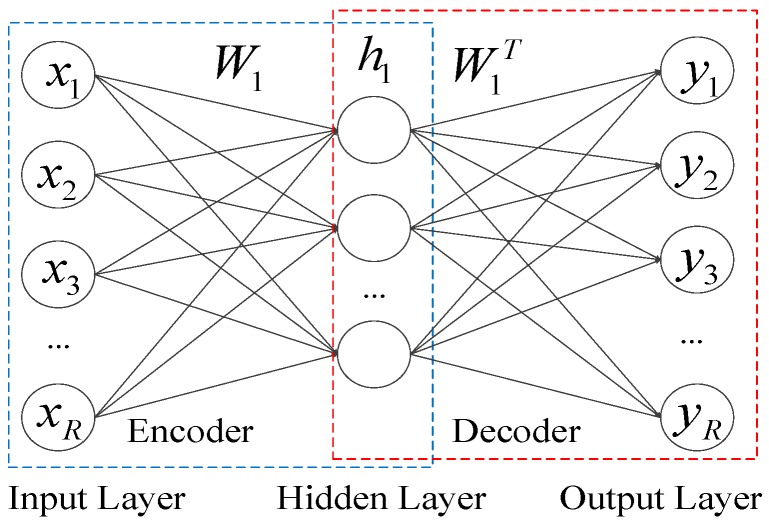
Auto-encoder on the first hidden layer of the DNN.

The AE is a three-layer unsupervised neural network comprising an input layer, a hidden layer, and an output layer. The input layer and the hidden layer are connected by the coding network, while the hidden layer and the output layer are connected by the decoding network. As shown in Figure 2, the input layer equals to the output layer. The AE can convert the input data into a more abstract feature space via a coding network, and coded vectors can also be reconstructed via the decoding network as an approximation of the input data.

Given an unlabeled dataset $x=\{x_{1},x_{2},\cdots ,x_{R}\}$, where R is the number of the input neuron of the AE on the first layer of a DNN, the encoding process can be described as follows:

(1) $$h_{1}=f_{\theta_{1}}(x)=\sigma (W_{1}x+b_{1})$$

where $f_{\theta_{1}}$ is the activation function used in the encoder network, $W_{1}$ is the weight matrix between the input layer and the hidden layer of $AE_{1}$, $b_{1}$ is the bias vector generated by the encoder network, $\theta_{1}=[W_{1},b_{1}]$ is the connection parameter between the input layer and the hidden layer and σ is the sigmoid function and a common choice for the activation function, depicted in Equation (2):

(2) σ(z)=1/[1+exp(−z)]

Then, $h_{1}$ is used as the input of $AE_{2}$ to train the network parameter $W_{2}$. The coded vector $h_{2}$ is obtained as the feature extracted on the second layer. This process is repeated until the Nth layer to train the network parameter $W_{N}$. Thus, $h_{N}$ can be obtained as the feature extracted on the Nth layer of DNN. For convenience, we use $W=[W_{1},W_{2},\cdots ,W_{N}]$, and $b=[b_{1},b_{2},\cdots ,b_{N}]$ to denote the network parameters of DNN, where $W_{n}\;(n=1,2,\cdots ,N)$ denotes the weight matrix on the nth layer of DNN, and $b_{n}\;(n=1,2,\cdots ,N)$ denotes the bias on the nth layer of DNN.

Similarly, the reconstruction process can be obtained via a decoder network as follows:

(3) $$y_{1}=g_{\theta_{1}{}^{T}}(h_{1})=\sigma (W_{1}{}^{T}h_{1}+d_{1})$$

where $y_{1}$ is the reconstructed data generated by the decoder function $g_{\theta_{1}{}^{T}}$, σ is the activation function of the decoder process, $W_{1}{}^{T}$ represents the weight matrix between the hidden layer and the output layer of the decoder network, $h_{1}$ represents the output of encoding process, and $d_{1}$ is the bias vector generated by the decoder process.

The use of AE pre-training is to optimize the network parameters $\theta_{1}=[W_{1}\;b_{1}]$ by minimizing the reconstruction described in Equation (4):

(4) $$J(x,y;\;W_{1},b_{1})=\frac{1}{M}{\left\|y-x\right\|}^{2}$$

where x denotes the input of $AE_{1}$ and y denotes the output of $AE_{1}$, and M denotes the number of training samples.

For the training of DNN, the gradient descent algorithm is used for parameter optimization, and the network parameter-updating process can be formulated in Equations (5) and (6):

(5) $$W_{1,l+1}=W_{1,l}-\alpha \frac{\partial }{\partial W_{1}}J(x,y;W_{1},b_{1}),l=1,2\cdots L$$

(6) $$b_{1,l}=b_{1,l}-\alpha \frac{\partial }{\partial b_{1}}J(x,y;W_{1},b_{1}),l=1,2\cdots L$$

where α is the learning rate, L is the maximum number of iterations for the back-propagation algorithm, $\frac{\partial }{\partial W_{1}}J(x,y;W_{1},b_{1})$ and $\frac{\partial }{\partial b_{1}}J(x,y;W_{1},b_{1})$ are the gradient descent direction. 

The DNN pre-training process is completed through unsupervised training layer by layer, and the features on each layer can be roughly extracted. A Softmax classifier is add to the top layer of DNN. $h_{N}$ is used as the input, and the labeled data set {1,2,…,S} is used as the output to train a Softmax classifier. Given an observation sample $x(m)=[x_{1}(m),x_{2}(m),\cdots x_{R}(m)]$ at time m, x(m) is used as the input of DNN to obtain its feature $h_{N}(m)$ extracted on the Nth layer. $h_{N}(m)$ is then used as the input of the well-trained Softmax classifier to obtain the category label of x(m) in Equations (7) and (8):

(7) $$label(m)=\underset{s=1,2\cdots ,S}{\arg\max}\{p(label(m)=s|x(m);\phi )\}$$

where p(lable(m)=s|x(m);ϕ) is the sth argument of the likelihood function vector $h_{\phi }(x(m))$ defined in Equation (8):

(8) $$h_{\phi }(x(m))=\left[\begin{array}{c} p(label(m)=1\;|x(m);\phi )\\ p(label(m)=2|x(m);\phi )\\ \vdots \\ p(label(m)=S|x(m);\phi ) \end{array}\right]=\frac{1}{\sum_{s=1}^{S}e^{\phi_{s}^{T}x(m)}}\left[\begin{array}{c} e^{\phi_{1}^{T}x(m)}\\ e^{\phi_{2}^{T}x(m)}\\ \vdots \\ e^{\phi_{S}^{T}x(m)} \end{array}\right]$$

where, $\phi =[\phi_{1},\phi_{2},\cdots ,\phi_{S}]$ is the model parameter of the Softmax classifier. The model parameters can also be optimized by minimizing the cost function defined in Equation (9):

(9) $$J(\phi )=-\frac{1}{M}[\sum_{m=1}^{M}\sum_{s=1}^{S}1\{label(m)=s\}\log\frac{e^{\phi_{s}^{T}x(m)}}{\sum_{s=1}^{S}e^{\phi_{s}^{T}x(m)}}]$$

where 1{•} is the indicated function.

Third, we fine-tune the network parameter of DNN. Once the Softmax classifier is added to the top layer of DNN, the labels of some observation samples can be used for reverse fine-tuning of DNN, which are shown as Equations (10) and (11):

(10) $$\theta_{}^{}=\theta -\alpha_{}\frac{\partial E(\theta_{}^{})}{\partial \theta }$$

(11) $$E(\theta_{}^{})=\min\frac{1}{M}\sum J(\phi |h_{N};L;\theta )$$

where L is the known label set, α is the learning rate of the reverse fine-tuning process, and θ can be calculated by Equations (5) and (6).

## 3. Differential Geometric Feature Fusion-Based Deep Neural Network Fault Diagnosis Method

As introduced above, although a frequency-type fault can be well recognized in the frequency domain, it is difficult to achieve online diagnosis, since the fault feature extracted in the time domain is usually not significant. To ensure the accuracy of online diagnosis, it is necessary to diagnose such faults in the time domain with an advanced feature extraction method. For this goal, abnormal frequency feature should be characterized in the time domain by effectively mining the dynamic trend information. This section first analyzes frequency-type faults, and then describes in detail the feature extraction and fault diagnosis methods proposed in this paper.

### 3.1. Frequency-Type Fault Analysis

**Figure 3:**
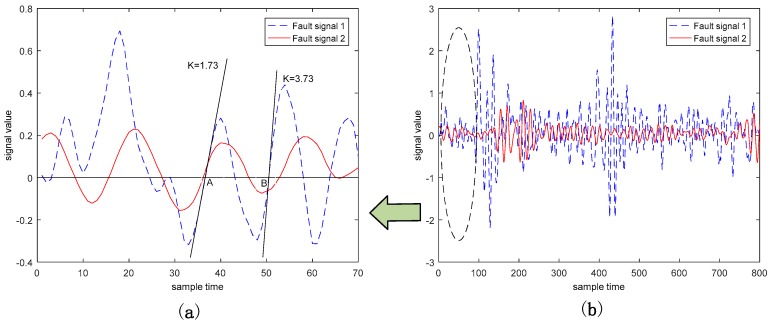
Slope features of the abnormal signal. (**a**) is the enlarged part of these two abnormal signals circled in (**b**).

Since the bearing data is a periodic vibration signal, there are a large amount of zero-crossing points in the abnormal signal defined by the difference between fault data and normal data. These abnormal signals can be seen as a kind of frequency-type fault, since frequent zero-crossing points appear in the abnormal signal, but the slopes or curvatures at these zeros-crossing points are not zero, as shown in Figure 3. On the other hand, the diagnosis mechanism of most existing fault diagnosis methods is to confirm whether the there is a non-zero difference between the feature extracted from the fault data and the feature extracted from the normal data. Thus, it is difficult to achieve an accurate online diagnosis of the frequency-type fault by only extracting the feature involved in the amplitude data. Features extracted from the slope and curvature data can be helpful for abnormal detection at zero-crossing points when the feature extracted from the amplitude data fails to achieve a satisfying fault diagnosis result.

**Figure 4:**
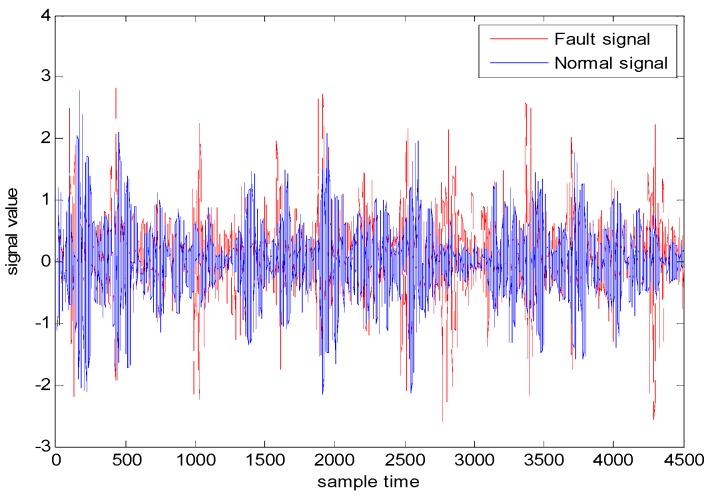
Normal and fault data in the time domain.

**Figure 5:**
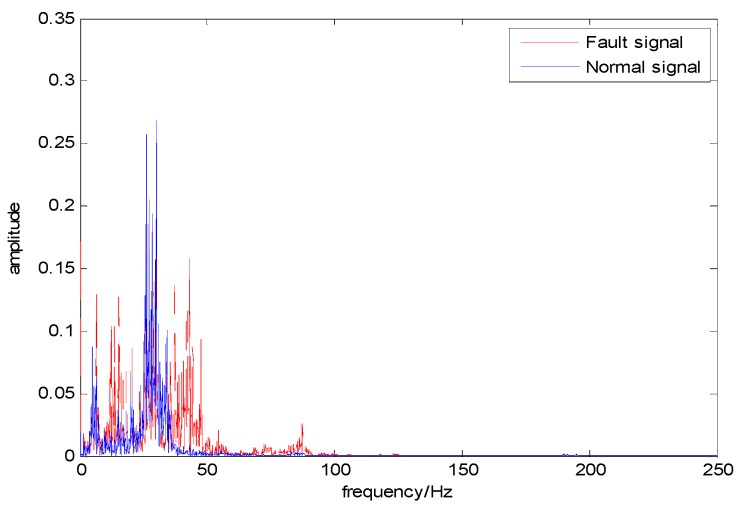
Normal and fault data in the frequency domain.

As can be seen from Figure 3, points “A” and “B” are the zero-crossing points of fault signal 1, called frequency-type faults. Both their amplitudes are 0, which makes the fault features involved in these zero-cross points not well characterized in the time domain, and the diagnosis effect, when based simply on the amplitude information, can hence be greatly reduced [27]. However, we can clearly distinguish these two types of fault data by using the slopes of these two fault signals (1.73 and 3.73, respectively). Figure 4 and Figure 5 illustrate the normal bearing data and the fault bearing data in the time domain and in the frequency domain, respectively, as examples, where the blue solid line represents the normal data, and the red dash-dotted line represents the fault data. It is obvious that, with the significant fault feature in the frequency spectral, simply extracting the feature involved in the frequency data can be used for effectively discriminating the health normal data and the fault data in the frequency domain, rather than distinguishing the normal data and the fault data in the time domain. Therefore, some scholars detect such faults through DL in the frequency domain. However, the diagnosis in the frequency domain cannot guarantee real-time performance, which is the primary requirement of the health monitoring of actual industrial systems. This real-time requirement of fault diagnosis can minimize security risks, since it can provide necessary information for the remaining useful life prediction of the mechanical device.

**Remark** **2.** *From Figure 4 and Figure 5, we can further understand the concept of a frequency-type fault: the fault feature involved in the frequency spectra is similar to that extracted from the time-domain amplitude data.*

In general, when the amplitude of the fault data and the normal data are equal but the slopes are different, there must be a fault occurring. In this case, differential geometric properties, such as the slope and curvature, can be used to characterize the dynamic trend, which is helpful for feature extraction of the frequency-type faults. The DNN method based on the differential geometric feature fusion proposed in this paper can provide an efficient means of online fault diagnosis by extracting potential features involved in frequency-type fault data to achieve accurate and real-time diagnosis in the time domain for frequency-type faults. 

### 3.2. Differential Geometric Feature Fusion-Based Deep Neural Network-Based Online Fault Diagnosis Methods

This section is divided into three parts to introduce the DGFFDNN-based online fault diagnosis method: multimodal differential feature extraction, multimodal feature fusion, and real-time online diagnosis of the frequency-type fault. The complete DGFFDNN-based online fault diagnosis algorithm is shown as follows.

#### 3.2.1. Multimodal Differential Feature Extraction

The first step of the DGFFDNN-based online fault diagnosis algorithm proposed in this paper is to extract multimodal differential features involved in the data by using a stacking AE. The multimodal feature extraction algorithm is as follows:

**Step 1:** Obtaining data that characterize the differential geometric features of the raw data. Therefore, the slope and curvature values of the historical raw data are calculated in Equations (12) and (13), respectively:

(12) $$x^{\prime }(m)=\frac{x(m+1)-x(m)}{T},\;(m=1,2,\cdots M-1)$$

(13) $$x^{\prime \prime }(m)=\frac{x\prime (m+1)-x\prime (m)}{T},\;(m=1,2,\cdots M-2)$$

where T is the sampling interval, and $x,\;x^{\prime }\;\mathrm{and}\;x^{\prime \prime }$ are the datasets corresponding to the raw magnitude data, the slope data, and the curvature data, respectively.

**Step 2**: Training the DNN model using historical data $x,\;x^{\prime }\;\mathrm{and}\;x^{\prime \prime }$.

Constructing three DNN networks with Equation (14), and initialize the training parameters of $DNN_{1}$, $DNN_{2}$, and $DNN_{3}$, respectively:

(14) $$\left\{ \begin{array}{l} \left[Net,Tr]=feedforward(\theta ;H_{1},H_{2},\ldots ,H_{N_{1}};x\right)\\ \left[Net^{\prime },Tr^{\prime }]=feedforward(\theta_{}^{\prime };H_{1}{}^{\prime },H_{2}{}^{\prime },\ldots ,H_{N_{2}}{}^{\prime };x^{\prime }\right)\\ \left[Net^{\prime \prime },Tr^{\prime \prime }]=feedforward(\theta_{}^{\prime \prime };H_{1}{}^{\prime \prime },H_{2}{}^{\prime \prime },\ldots ,H_{N_{3}}{}^{\prime \prime };x^{\prime \prime }\right) \end{array}\right.$$

where “feedforward” is the MATLAB function to generate a multilayer neuron network; $N_{1}$ is the number of hidden layers of $DNN_{1}$; $H_{n}\;(n=1,2,\cdots ,N_{1})$ is the number of neurons in the nth hidden layer of $DNN_{1}$; $\theta_{}^{}=\{W_{},\;b_{}\}$ is the network parameter, where $W_{}$ and b are the weight matrix and bias vector of $DNN_{1}$, respectively; Tr is the network parameter configuration. The number of input neurons of DNN can be determined by Equation (15):

(15) $$M_{}=size\left(x,\;2\right)$$

The parameters of $DNN_{1}$ are initialized by Equations (16) and (17):

(16) $$W_{}=rand\left(H_{},M_{}\right)$$

(17) $$b_{}=zeros(H_{},1)$$

where $H=H_{1}+H_{2}+\cdots +H_{N_{1}}$.

Unsupervised layer-by-layer feature extraction is implemented by the training process of $DNN_{1}$ shown in Equation (18):

(18) $$\left\{ \begin{array}{l} h_{1}=f_{\theta_{1}^{}}(x)=\sigma (W_{1}\cdot x+b_{1})\\ h_{2}=f_{\theta_{2}^{}}(h_{1})=\sigma (W_{2}\cdot h_{1}+b_{2})\\ \;\vdots \\ h_{N_{1}}=f_{\theta_{N}^{}}(h_{N_{1}-1})=\sigma (W_{N_{1}}\cdot h_{N_{1}-1}+b_{N_{1}}) \end{array}\right.$$

The feature $h_{N_{1}}$ on the top layer of $DNN_{1}$ can be extracted by this layer-by-layer process, as shown in Figure 2.

DNN_2_ and DNN_3_ networks are similarly built, using Equations (15)–(18). The multimodal differential features corresponding to the original data, the slope data, and the curvature data can be extracted with Equation (19):

(19) $$\left\{ \begin{array}{l} h_{N_{1}}=f(x_{})=\sigma (W_{N_{1}}h_{N_{1}-1}+b_{N_{1}})\\ h_{N_{2}}{}^{\prime }=f(x_{}{}^{\prime })=\sigma (W_{N_{2}}{}^{\prime }h_{N_{2}-1}{}^{\prime }+b_{N_{2}}{}^{\prime })\\ h_{N_{3}}{}^{\prime \prime }=f(x_{}{}^{\prime \prime })=\sigma (W_{N_{3}}{}^{\prime \prime }h_{N_{3}-1}{}^{\prime \prime }+b_{N_{3}}{}^{\prime \prime }) \end{array}\right.$$

where $h_{N_{1}}{}^{}$ is the deep feature of the raw magnitude data x; $h_{N_{2}}{}^{\prime }$ is the deep feature of the slope data $x_{}{}^{\prime }$; $h_{N_{3}}{}^{\prime \prime }$ is the deep feature of the curvature data $x_{}{}^{\prime \prime }$.

A Softmax classifier is then added to the top layer of DNN.

The training errors of $DNN_{1}$, $DNN_{2}$, and $DNN_{3}$, corresponding to the magnitude data, slope data, and the curvature data, are calculated with Equation (20):

(20) $$\left\{ \begin{array}{l} J_{1}(x,L;\;W_{},b_{})=\frac{1}{M}{\left\|P-L\right\|}^{2}\\ J_{2}(x^{\prime },L^{\prime };\;W^{\prime },b^{\prime })=\frac{1}{M-1}{\left\|P^{\prime }-L^{\prime }\right\|}^{2}\\ J_{3}(x^{\prime \prime },L^{\prime \prime };\;W^{\prime \prime },b^{\prime \prime })=\frac{1}{M-2}{\left\|P^{\prime \prime }-L^{\prime \prime }\right\|}^{2} \end{array}\right.$$

where $P,\;P^{\prime }\;\mathrm{and}\;P^{\prime \prime }$ are the predicted likelihood function values computed by Equation (8), corresponding to $DNN_{1}$, $DNN_{2}$, and $DNN_{3}$, respectively; $L,\;L^{\prime }\;\mathrm{and}\;L^{\prime \prime }$ are the known labels of $x,x^{\prime }\;\mathrm{and}\;x^{\prime \prime }$, respectively.

The gradient descent method is used for parameter optimization, and the specific updating process of network parameters can be performed with Equations (5) and (6). When the reconstruction error reaches a minimum after the network parameters are fine-tuned, it means that the DNN parameter is well trained, and $h_{N_{1}}$, $h_{N_{2}}^{\prime }$ and $h_{N_{3}}^{\prime \prime }$ are the multimodal features extracted from the raw magnitude data, the slope data, and the curvature data, respectively.

#### 3.2.2. Multimodal Differential Feature Fusion

As illustrated in Figure 5, the slope of different fault data may also be equal; that is, we cannot effectively classify the different faults by simply using the slope feature, which may be equal for different fault data. Thus, fusing a multimodal differential feature to obtain a new fused feature is necessary to mine the dynamic trend in the time domain, which is an essential step of feature extraction for a frequency-type fault. In this paper, the multimodal differential feature is integrated to capture the frequency feature of abnormal signal in the time domain and fused by a stacked form to obtain a new fused feature with a higher dimension. The features $h_{N_{1}},h_{N_{2}}{}^{\prime }\;\mathrm{and}\;h_{N_{3}}{}^{\prime \prime }$ extracted from the above three well-trained DNN models can be fused to obtain a new feature vector, F, with Equation (21):

(21) $$F=[F_{1},F_{2},F_{3}]$$

where $F_{1}=h_{N_{1}}$, $F_{2}=h_{N_{2}}$ and $F_{3}=h_{N_{3}}$ are the multimodal differential features extracted from $DNN_{1}$, $DNN_{2}$, and $DNN_{3}$, respectively.

**Figure 6:**
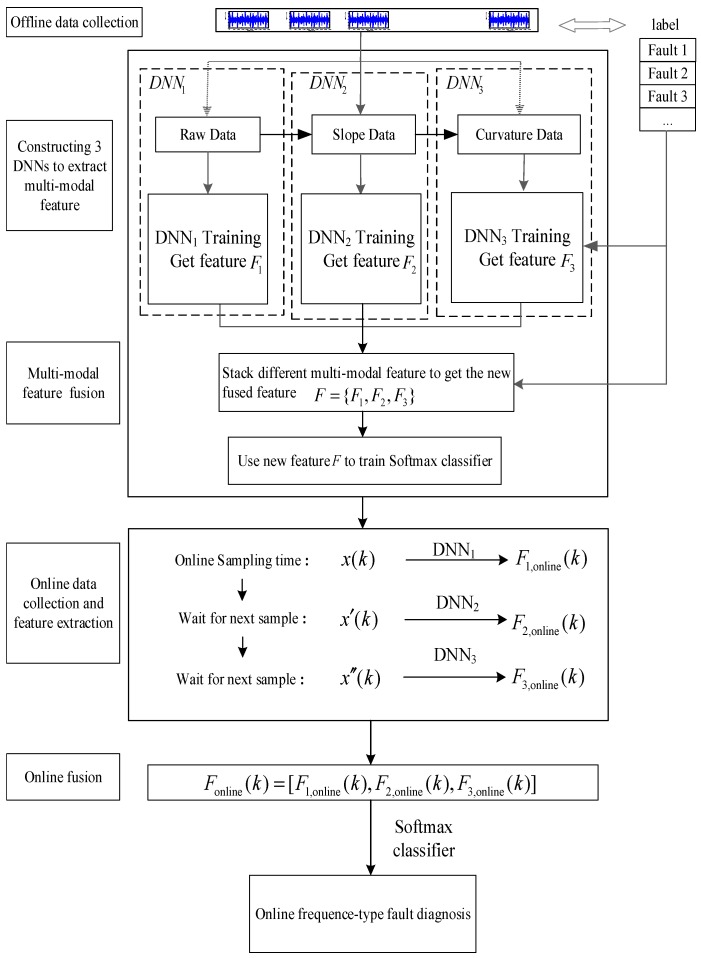
Feature fusion process.

The whole feature fusion process is shown in Figure 6.

**Figure 7:**
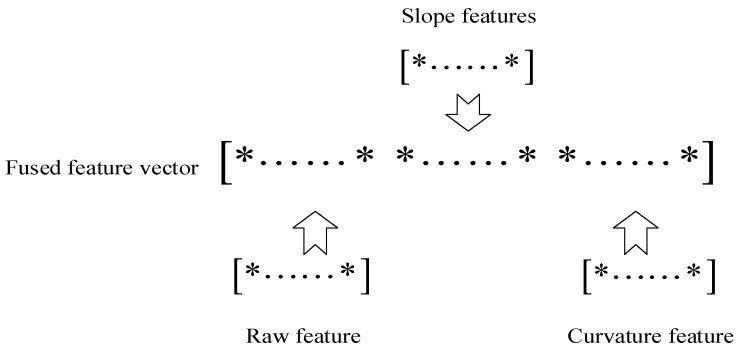
Schematic diagram to obtain a fused feature vector.

A fused feature vector can be obtained by combining the multimodal features (slope, raw and curvature features) extracted by these three DNNs, which is illustrated in Figure 7.

In the final step, we use the fused feature as the input, and the fault label of each sample as the output, to train the Softmax classifier.

#### 3.2.3. Online Diagnosis

**Figure 8:**
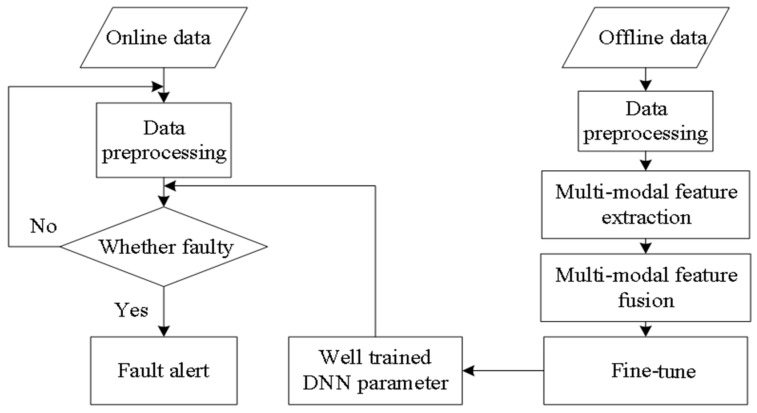
Differential geometric feature fusion-based DNN (DGFFDNN)-based diagnosis for frequency-type faults.

Real-time diagnosis uses well-trained DGFFDNN parameters to identify the faults involved in online data. The frame of DGFFDNN-based fault diagnosis for frequency-type faults is illustrated in Figure 8.

The online fault diagnosis process is as follows:

**Step 1:** Extracting online multimodal differential features.

When the online observation at time k, denoted as $x_{online}(k)$, is available, the well-trained ${DNN}_{1}$ is used to extract the amplitude feature involved in the online raw data in Equation (22):

(22) $$h_{N_{1},online}(k)=G(Net,\;Tr,x_{online}(k))$$

where the function G is used to illustrate the fact that the online amplitude feature is the output of the trained network ${DNN}_{1}$ when $x_{online}(k)$ is the input of the network.

Then, waiting for the observation at time k+1 until $x_{online}(k+1)$ is available, the slope at time k can be computed first in Equation (23):

(23) $$x_{online}^{\prime }(k)=\frac{x_{online}(k+1)-x_{online}(k)}{T}$$

Similar to Equation (23), the slope feature can be extracted from the well-trained ${DNN}_{2}$ in Equation (24):

(24) $$h_{N,online}^{\prime }(k)=G^{\prime }(Net^{\prime },\;Tr^{\prime },x_{online}^{\prime }(k))$$

Waiting for the observation at time k+2 until $x_{online}(k+2)$ is available, the curvature at time k can be computed in Equations (25) and (26):

(25) $$x_{online}^{\prime }(k+1)=\frac{x_{online}(k+2)-x_{online}(k+1)}{T}$$

(26) $$x_{online}^{\prime \prime }(k)=\frac{x_{online}^{\prime }(k+1)-x_{online}^{\prime }(k)}{T}$$

The curvature feature can also be extracted from the well trained ${DNN}_{3}$ in Equation (27):

(27) $$h_{N,online}^{\prime \prime }(k)=G^{\prime \prime }(Net^{\prime \prime },\;Tr^{\prime \prime },x^{\prime \prime }(k))$$

**Step 2:** Fusing multimodal differential features for online data.

The multimodal differential feature at time k is used to obtain the fused feature in Equation (28):

(28) $$F_{online}(k)=[F_{1,online}(k),F_{2,online}(k),F_{3,online}(k)]$$

where $F_{1,online}(k)=h_{N_{1}}(k),\;F_{2,online}(k)=h^{\prime }{}_{N_{2}}(k)\;\mathrm{and}\;F_{3,online}(k)=h_{N_{1}}^{\prime \prime }(k)$.

**Step 3:** Performing online diagnosis for frequency-type faults.

According to the design of the Softmax classifier, the class that maximizes the likelihood function is the online diagnosis result of the online samples $x_{online}(k)$, shown in Equations (29) and (30):

(29) $$h_{\phi }(x_{online}(k))=\left[\begin{array}{c} p(label(k)=1\;|x_{online}(k);\phi )\\ p(label(k)=2|x_{online}(k);\phi )\\ \vdots \\ p(label(k)=S|x_{online}(k);\varphi ) \end{array}\right]=\frac{1}{\sum_{s=1}^{S}e^{\phi_{s}^{T}x_{online}(k)}}\left[\begin{array}{c} e^{\phi_{1}^{T}x_{i}}\\ e^{\phi_{2}^{T}x_{online}(k)}\\ \vdots \\ e^{\phi_{s}^{T}x_{online}(k)} \end{array}\right]$$

(30) $$result(k)=\underset{s=1,2\cdot \cdot \cdot ,S}{\arg\max}\{p(label(k)=s|h_{\phi }(x_{online}(k));\phi )\}$$

where result(k) is the fault diagnosis result of the online data $x_{online}(k)$.

**Figure 9:**
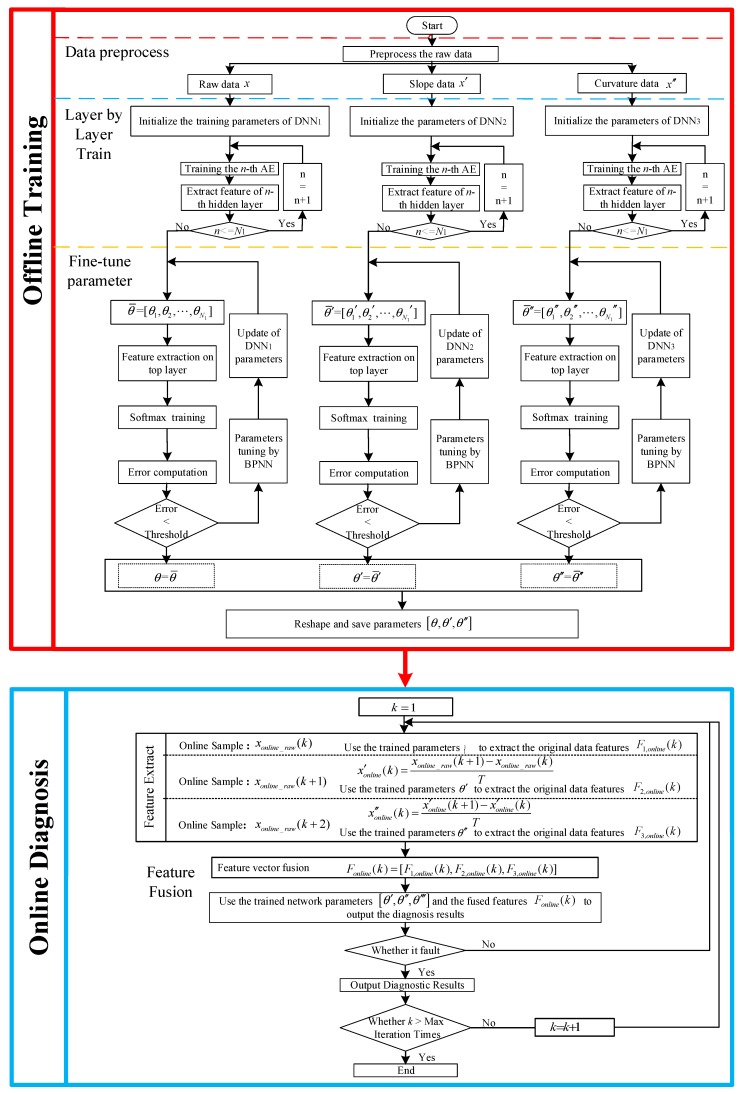
A fault diagnosis flowchart based on DGFFDNN.

The flow chart of the proposed DGFFDNN-based fault diagnosis algorithm for the frequency-type fault is shown in Figure 9.

## 4. Experiment and Analysis

Rolling bearing plays a crucial role in rotating machinery, which commonly suffers frequency-type faults. In this paper, a simulation study and a bearing case study were both illustrated to validate the efficiency of the DGFFDNN-based fault diagnosis method. The proposed method was compared with the DNN-based method without feature fusion.

### 4.1. Simulation Study

This paper aimed to effectively detect frequency-type faults in the time domain, which is difficult for traditional DL methods to identify different types of online faults in a mechanical system. This section validates the effectiveness of the proposed algorithm by simulating multiple sets of different fault-type test data. Analyses of three typical experiment scenes are illustrated in detail: different amplitudes with different frequencies, different amplitudes with the same frequency, the same amplitude with different frequencies. 

#### 4.1.1. Description of Simulation Experimental Data

**Figure 10:**
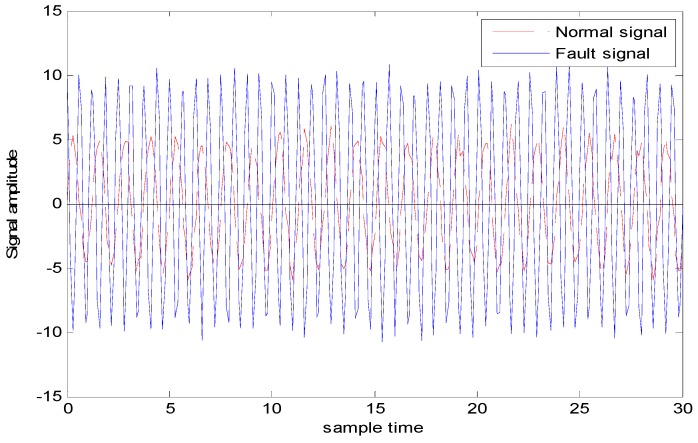
Normal and fault signals with different amplitudes and different frequencies.

The simulation data generation scheme is shown in Table 1, different amplitudes with the same frequency, the same amplitude with different frequencies). The generated observation data for case 1 (i.e., different amplitudes with different frequencies is shown in Figure 10 where the red line represents the normal observation, and the blue dashed line represents the fault observation.

To reduce the influence of randomness, the experiment was repeated 10 times. The DNN training uses a stochastic gradient descent method, and the maximum numbers of iterations of DNN in each layer were 1000, 800, and 1000 times, respectively. DNN’s pre-training initialization parameters are shown in Table 2.

#### 4.1.2. Analysis of Simulation and Experiment Results

**Figure 11:**
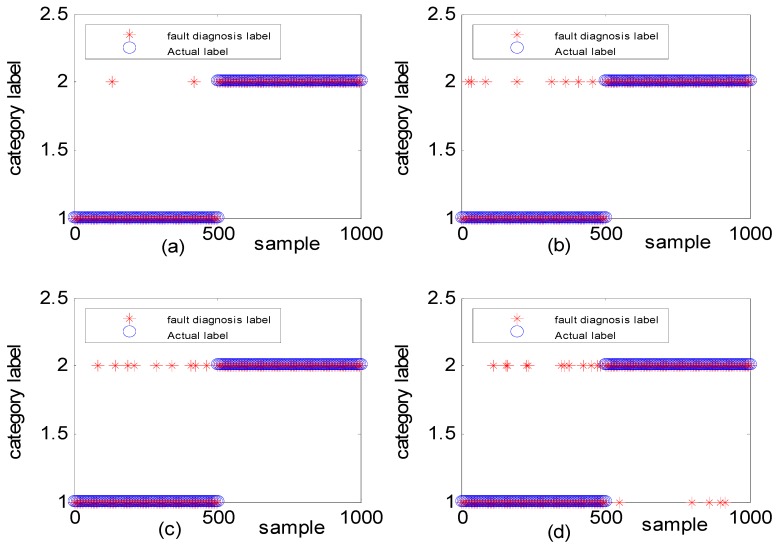
Simulation results for diagnosis of fault with different fault amplitudes and different fault frequencies: (**a**) DGFFDNN, (**b**) DNN, (**c**) DGFFBP, and (**d**) BP. A red star represents the fault diagnosis label of each online sample, and a blue circle represents the real label of each sample. At each sample time, the coincidence of a red circle and a blue star means that the online observation at this sample time is correctly diagnosed.

To verify the effectiveness of the algorithm, different types of simulation data were used to illustrate the experimental result. Figure 11 shows the fault diagnosis results corresponding to DGFFDNN, DNN, Differential geometry feature fusion-based back propagation (DGFFBP), and back propagation (BP), respectively, for experiment case 1: different amplitudes with different frequencies. The accuracies of DGFFDNN, DNN, DGFFBP, and BP are 98.4%, 94.24%, 92.36%, and 90.86%, respectively. 

**Figure 12:**
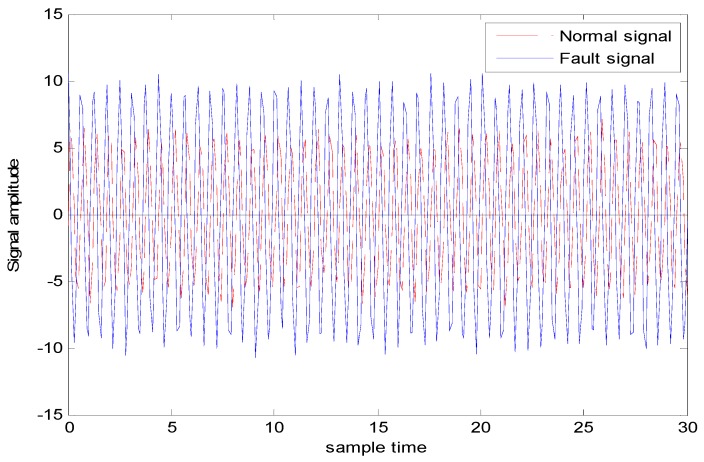
Normal and fault signals with different amplitudes and the same frequency and.

The generated observation data for case 2 (i.e., Different amplitudes with the same frequencies is shown in Figure 12, where the red dashed line represents the normal observation, and the blue line represents the fault observation.

**Figure 13:**
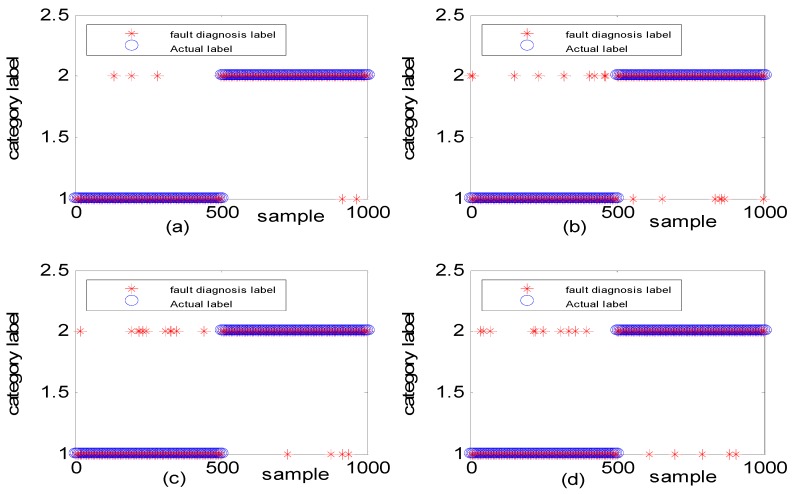
Simulation study for diagnosis of faults with the same frequency and different fault amplitudes: (**a**) DGFFDNN, (**b**) DNN, (**c**) DGFFBP, and (**d**) BP. A red star represents the fault diagnosis label of each online sample, and a blue circle represents the real label of each sample. At each sample time, the coincidence of a red circle and a blue star means that the online observation at this sample time is correctly diagnosed.

Figure 13 shows the fault diagnosis result corresponding to experiment case 2: different amplitudes with the same frequency. The accuracies of DGFFDNN, DNN, DGFFBP and BP are 94.34%, 92.01%, 90.69%, and 87.04%, respectively. 

**Figure 14:**
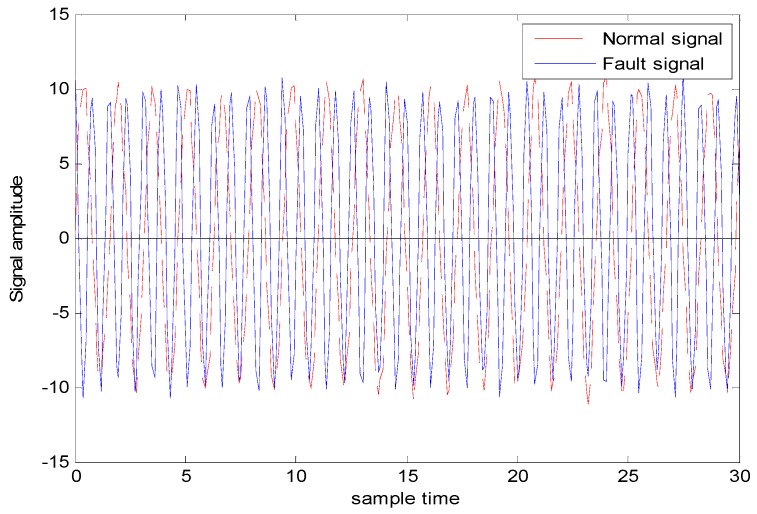
Normal and fault signals with the same amplitudes and different frequencies.

**Figure 15:**
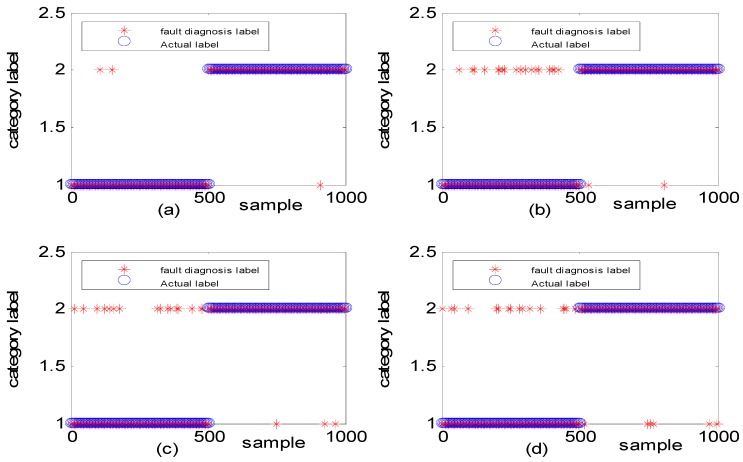
Simulation study for diagnosis of faults with different frequencies: (**a**) DGFFDNN, (**b**) DNN, (**c**) DGFFBP, and (**d**) BP. A red star represents the fault diagnosis label of each online sample, and a blue circle represents the real label of each sample. At each sample time, the coincidence of a red circle and a blue star means that the online observation at this sample time is correctly diagnosed.

The generated observation data for case 3 (i.e., the same amplitudes with different frequency is shown in Figure 14, where the red dashed line represents the normal observation, and the blue line represents the fault observation. Figure 15 shows the fault diagnosis result corresponding to experiment case 3: the same amplitudes with different frequencies. The accuracies of DGFFDNN, DNN, DGFFBP, and BP are 93.06%, 73.54%, 62.87%, and 54.36%, respectively. 

It can be easily seen that the diagnosis accuracy of DGFFDNN is higher than the traditional DNN, and the diagnosis accuracy of DGFFBP is higher than the traditional BP, which demonstrates that the differential geometry feature fusion-based method is an efficient means for diagnosing a frequency-type fault. It can also be concluded that DGFDNN is superior to the other three methods in real-time and accurate diagnosis for a frequency-type fault. 

It can be seen in Figure 11a, Figure 13a and Figure 15a that if the fault size is large in both a time domain and frequency domain, even a traditional shallow learning method can be used for a relatively satisfactory diagnosis result. In the case when the fault size was only large in amplitude, the traditional DNN can be used to achieve a relatively satisfactory diagnosis, which the traditional BP cannot. As for the case of the frequency-type fault, that is, the fault size is large in frequency but very small in amplitude, the accuracy of the traditional shallow learning method is only 54.36% which cannot meet the engineering requirement, and even the DNN can only achieve a diagnosis accuracy of 73.54%. However, the method proposed in this paper can greatly improve the diagnosis accuracy to a higher value of 93.06%, showing that DGFDNN is an efficient online diagnosis method for a frequency-type fault. 

Table 3 lists the fault diagnosis accuracy of the four fault diagnosis methods in three different experiment cases. It can be seen from Table 3 that the DGFFDNN method with an accuracy improvement of about 20% for experiment case 3 is greatly superior to other machine learning methods for the real-time diagnosis of typical frequency-type faults.

### 4.2. Case Study

To further verify the algorithm’s validity in engineering practice, the bearing experimental platform established by our research team and a benchmark rolling bearing test data set provided by Case Western Reserve University (CWRU) were both used to verify the effectiveness of DGFFDNN. We carried out an algorithm test on two datasets: (1) different fault diameter with the same fault type; and (2) different fault types with same fault diameters.

#### 4.2.1. Description of the Experimental Platform

**Figure 16:**
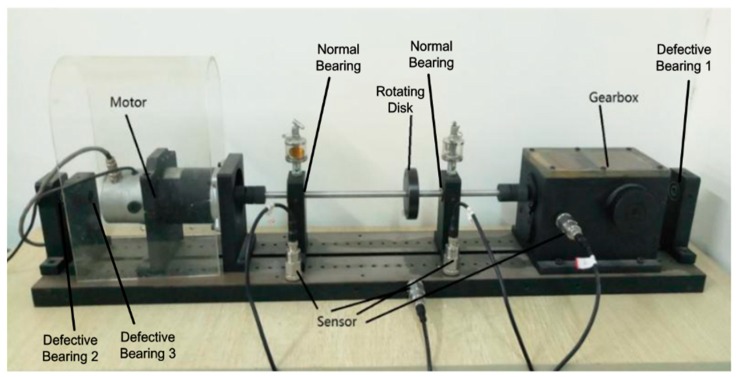
Experiment platform of bearing in Henan University.

The experimental dataset was the bearing data collected from our fault diagnosis test platform. Figure 16 displays the experimental platform established by the data-driven research team of Henan University. The experimental platform was comprised of a motor, three defective bearings, two normal bearings, a gearbox, a shaft, a rotating disc, and four sensors. Vibration data were collected using accelerometers, which were attached to the platform with magnetic bases.

The normal bearing installed on the shaft was replaced by a defective bearing with a given fault diameter to simulate different bearing faults, such as an inner race fault, an out race fault, and a ball fault. The normal gear installed in the gearbox was replaced with a defective gear with a given number of broken teeth to simulate a gearbox fault. Adjusting the stress balance on the rotating disc was used to simulate the shaft fault. In this paper, the only bearing fault data with no other concurrent fault was collected for the DGFFDNN algorithm test. The sampling frequency was 48 kHz.

**Remark** **3.** *Single-point bearing faults with given fault diameters of 0.007 inches, 0.014 inches and 0.021 inches were provided by a sail company, Kun Long Jia Chen. A convenient bearing replacing was required to simulate with different bearing fault data.*

**Remark** **4.** *There was no special unit, such as an additional motor, to simulate the experiment of load changing, so the vibration data was collected in the case when load was 0 horsepower (hp). Although the gearbox can be seen as a load, it is very small. According to the engineering criteria of Kun Long Jia Chen Company, varying loadings only affects the amplitude of the abnormal signal, and cannot affect the frequency of the abnormal signal. Therefore, the efficiency of the DGFFDNN-based frequency-type fault diagnosis method was not influenced.*

#### 4.2.2. Case Study Result Analysis

The DGFFDNN method proposed in this paper was applied to bearing fault diagnosis. There were 4500 samples under each data type, and four different data types to characterize the frequency fault types of rotating mechanical systems.

Three fault datasets and a normal dataset with 40,000 samples were collected to test the algorithm, with 40,000 samples for the DGFFDNN training, 2000 samples for the test. The fault data corresponded to the inner race fault with different fault sizes in the case when load was 0. The fault diameters of the defective bearing were 0.007 inches, 0.014 inches, and 0.021 inches, respectively. Four experiment cases were chosen with these data types having similar amplitude features but different frequency features.

**Figure 17:**
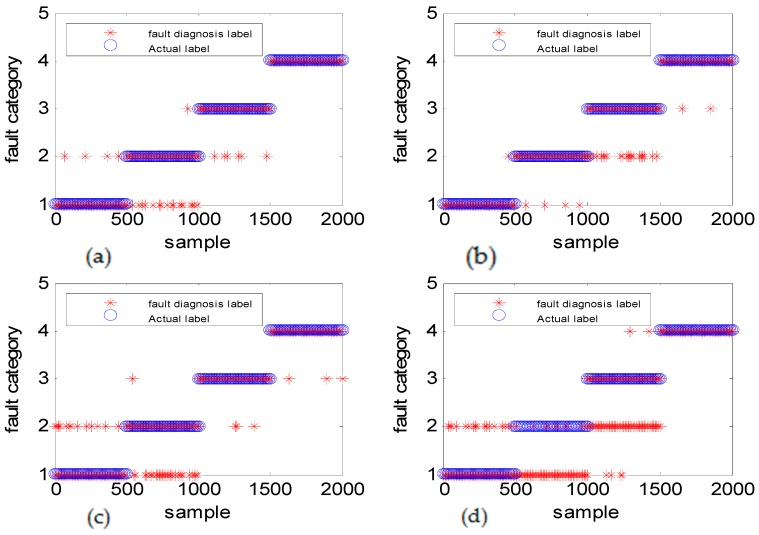
Case study results for diagnosis for different fault sizes: (**a**) DGFFDNN, (**b**) DNN, (**c**) DGFFBP, and (**d**) BP.

Figure 17 shows the diagnostic results of experiment case 1: the same fault size with different fault types. The accuracies of DGFFDNN, DNN, DGFFBP, and BP are 98.26%, 91.32%, 87.06%, and 80.28%, respectively. The DGFFDNN diagnosis result (Figure 17a) can distinguish different fault sizes well and is better than the other three methods, which provides very useful information for failure prediction maintenance and is effective in the fault diagnosis of the engineering application field.

**Figure 18:**
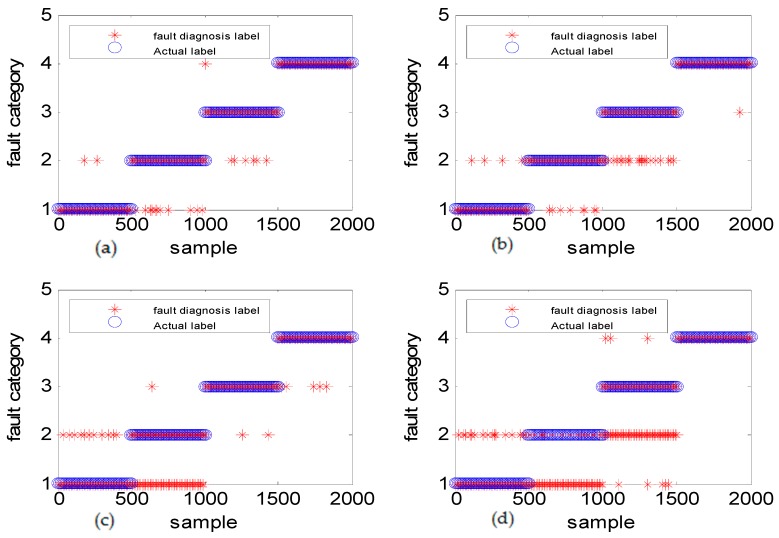
Case study for diagnosis of different types of fault with the same size of 0.007 inches: (**a**) DGFFDNN, (**b**) DNN, (**c**) DGFFBP, and (**d**) BP.

Figure 18 shows the diagnostic results of experiment case 2: the same fault size with different fault types where the used fault size was only 0.007 inches. The accuracies of DGFFDNN, DNN, DGFFBP, and BP are 97.52%, 88.50%, 86.34%, and 76.29%, respectively, demonstrating the DGFFDNN diagnosis method is better than the other three methods in effectively distinguishing different fault types when multiple faults occur in the rotation mechanical equipment.

#### 4.2.3. Benchmark Dataset Testing

**Figure 19:**
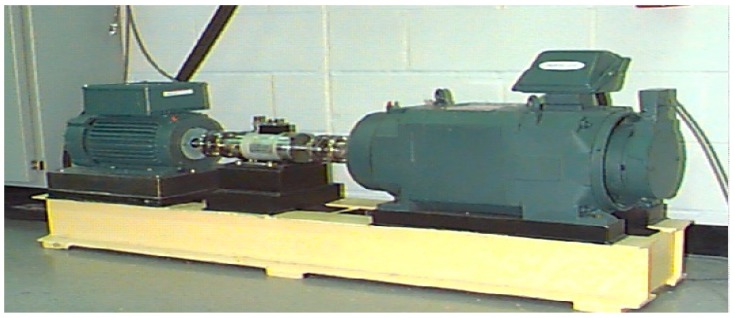
Experimental platform of the bearing from Case Western Reserve University (CWRU) [54].

In this section, data from the CWRU Bearing Data Center as a benchmark dataset were used to test this DGFFDNN diagnosis algorithm, since most studies use this dataset for fault diagnosis algorithm testing [54]. The experiment platform is shown in Figure 19. The CWRU Bearing Data Center provides free access to download bearing data from the following website [54]. 

Fault simulation experiments were conducted using a 2-hp reliance electric motor, and acceleration data were measured at locations that were near or remote from the motor bearings. Motor bearings were seeded with faults using electro-discharge machining (EDM). Faults with diameters ranging from 0.007 inches to 0.040 inches were introduced separately at the inner raceway, rolling element (i.e., ball), and outer raceway. Faulted bearings were reinstalled into the test motor, and vibration data were recorded for the motor load range of 0 hp to 3 hp (motor speeds ranging from 1720 rpm to 1797 rpm).

**Figure 20:**
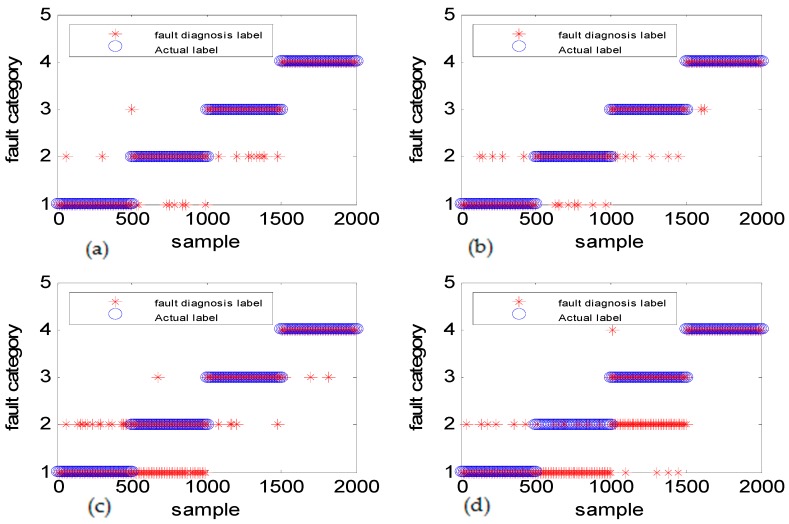
Benchmark test for the diagnosis of different fault types with the same size: (**a**) DGFFDNN, (**b**) DNN, (**c**) DGFFBP, and (**d**) BP. The number of samples used in the experiment was 487,384. To visualize the classification results, only part of the experimental results were displayed. The experiment used a normal dataset and three fault datasets. The three sets of fault data were the cases where the inner ring fault sizes were 0.007 inches, 0.014 inches, and 0.021 inches, respectively, when the load was 3 hp.

Figure 20 shows the diagnostic results of experiment case 1: the same fault size with the different fault types. The fault diameter was 0.07 inches, which was rather small. The accuracies of DGFFDNN, DNN, DGFFBP, and BP are 97.73%, 89.2%, 86.37%, and 60.24%, respectively. It can be clearly seen that the DGFFDNN diagnosis method (Figure 20a) is better than other three methods in distinguishing the fault size, which is very helpful for fault prognosis and maintenance.

**Figure 21:**
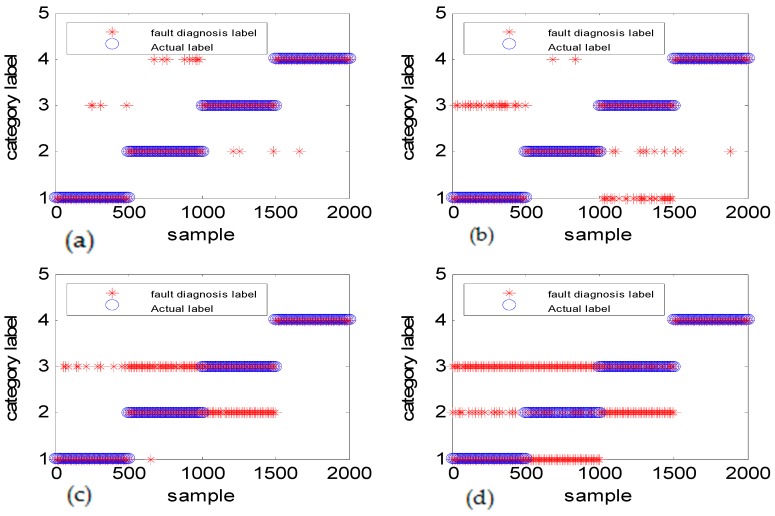
Benchmark test for the diagnosis of the same fault with different sizes: (**a**) DGFFDNN, (**b**) DNN, (**c**) DGFFBP, and (**d**) BP.

Figure 21 shows the diagnostic results of experiment case 2: the same fault type with different fault sizes. The accuracies of DGFFDNN, DNN, DGFFBP, and BP are 98.06%, 89.52%, 87.73%, and 73.56%, respectively, demonstrating the DGFFDNN diagnosis method (Figure 21a) can effectively diagnose multiple faults occurring in the rotation mechanical equipment.

From the above comparison, it can be concluded that the differential geometry feature fusion-based DNN method can be validated by the simulation study as well as the case study. Table 4 lists the diagnosis accuracy for the case study. It can be seen from Table 4 that the diagnostic accuracy of the proposed method is an effective fault diagnosis method for rotation mechanical equipment.

**Figure 22:**
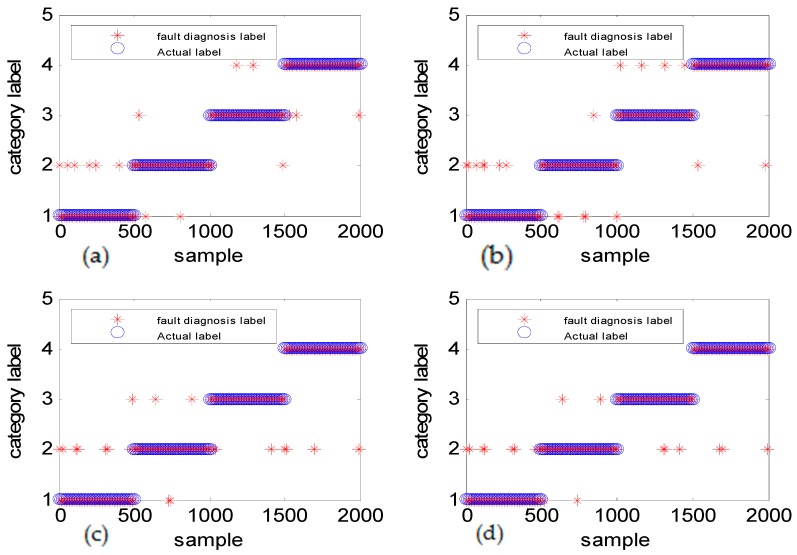
DNN diagnostic results using FFT as a preprocessing tool. (**a**) DGFFDNN, (**b**) DNN, (**c**) DGFFBP, and (**d**) BP.

Literature [40] studied the DNN-based fault diagnosis method for rolling bearing in the frequency domain. For the purpose of performance comparison, the diagnosis result of the algorithm proposed in [40] is also shown in Figure 22; the window size of FFT was set to be 500 samples. Table 4 list the comparison of DGFDNN algorithm and the algorithm proposed in [40].

**Remark** **5.** *Although the “fault classification” accuracy is higher than 99%, it is still not a suitable method in the engineering field of fault diagnosis, since fast Fourier transform (FFT) is required as a preprocessing tool that leads to a non-real time diagnosis method. In this paper, the frequency-type fault is diagnosed online in the time domain, and the diagnosis accuracy is higher than 97.63%, which is suitable for engineering applications.*

## 5. Conclusions and Future Work

In real-time and accurate are the primary performance requirements of the fault diagnosis method for safety security for key machinery in an automatic system. However, the DNN with FFT as a preprocessing tool is unable to achieve an online real-time fault diagnosis, since frequency spectra rather than observation at a sample time is the input of DNN. The main innovation of this paper is to develop a good dynamic trend capturing method in the time domain by using the methodology of DNN feature fusion to extract more accurate fault features, and fusing multimodal dynamic differential features. 

Compared with the DNN-based fault diagnosis method, the proposed DGFFDNN method with higher accuracy in achieving an online real-time diagnosis for rotating machinery has been well presented and experimentally validated using the diagnosis of the rolling bearing case study as well as the simulation study.

On a basis of the current work, further research can be dedicated to online diagnosis of early concurrent faults. A residual useful life prognosis based on early diagnosis of rotating machinery is another promising research direction.

## Figures and Tables

**Table 1:** Simulation of the data generation scheme.

Different Experimental Cases	Sampling Interval	Normal Observation	Fault Observation
Different amplitudes with different frequencies	0.1	$y_{1}=5\times \sin(5\times t)+\mathrm{awgn}(k)$	$y_{2}=10\times \cos(10\times t)+\mathrm{awgn}(k)$
Different amplitudes with the same frequencies	0.1	$y_{1}=6\times \sin(10\times t)+\mathrm{awgn}(k)$	$y_{2}=10\times \cos(10\times t)+\mathrm{awgn}(k)$
Different frequency with the same amplitudes	0.1	$y_{1}=10\times \sin(4\times t)+\mathrm{awgn}(k)$	$y_{2}=10\times \cos(8\times t)+\mathrm{awgn}(k)$

**Table 2:** DNN model parameters.

Training Parameter	$DNN_{1}$	$DNN_{2}$	$DNN_{3}$
Hidden layers	6	4	5
Number of neurons	500/400/200/100/50/10	500/100/50/20/10	500/200/100/50/20/10
Max number of epochs	1000	1000	1000
Learning rate	0.01	0.02	0.01

**Table 3:** Simulation study on accuracies of different fault diagnosis methods.

Data	DGFFDNN	DNN	DGFFBP	BP
Different amplitudes with different frequencies	98.40	94.24	92.36	90.86
Same frequency with different amplitudes	94.34	92.01	90.69	87.04
**Same amplitudes with different frequencies**	**93.06**	**73.54**	**62.87**	**54.36**

**Table 4:** Case study: accuracies of different fault diagnosis methods. (The unit of the fault diameter is in inch).

	DGFFDNN	DNN	DGFFBP	BP	DNN with FFT
**Henan University Bearing Platform**					
Different fault diameters (0.007, 0.014, 0.021, 0)	98.54%	90.14%	88.16%	80.13%	99.37%
Different fault types (inner race, ball, out race, normal)	97.63%	89.53%	86.42%	70.84%	99.24%
**Case Western Reserve University Bearing Platform**					
Different fault diameters (0.007, 0.014, 0.021, 0)	97.73%	89.52%	86.37%	60.24%	99.16%
Different fault types (inner race, ball, out race, normal) **Online diagnosis**	98.06% Yes	89.52% Yes	87.73% Yes	73.56% Yes	99.22% No
